# The interest of intranasal clonidine in the prevention of perioperative children’s anxiety: a prospective randomized trial

**DOI:** 10.11604/pamj.2023.46.37.40310

**Published:** 2023-09-25

**Authors:** Mariem Keskes, Nouha Amouri, Salma Ketata, Rahma Derbel, Maha Charfi, Imen Zouche, Moncef Sallemi, Moez Elloumi, Hichem Chikhrouhou

**Affiliations:** 1 Department of Anesthesiology and Intensive Care Unit, Habib Bourguiba University Hospital, Sfax, Tunisia,; 2 Department of Hematology, Hedi Chaker Hospital, Sfax, Tunisia,; 3 Department of Oto-Rhino-Laryngology, Habib Bourguiba University Hospital, Sfax, Tunisia

**Keywords:** Perioperative anxiety, intranasal clonidine, children’s premedication

## Abstract

**Introduction:**

perioperative anxiety in children may lead to psychological and physiological side effects. Clonidine is in increasing use in the pediatric population as an anxiolytic, sedative, and analgesic because of its central alpha2-adrenergic agonist effect. Our study aimed to evaluate the effect of clonidine in the prevention of perioperative children´s anxiety.

**Methods:**

we conducted a prospective controlled randomized double-blinded clinical trial including children aged between 2 and 15 years undergoing tonsillectomy surgery. The patients were randomly allocated to receive either an intranasal dose of clonidine (4 μg/kg) (clonidine group) or an equal volume dose of saline solution (control group) 30 minutes before entering the operating room. The level of anxiety assessed using the m-YPAS score was recorded before premedication, at the time of parent-child separation, and at the time of installation in the operating room. Acceptance of premedication, degree of sedation on entering the operating room as well as agitation on awakening, and sedation on arrival post-anesthesia care unit were noted. Adverse effects were recorded during the surgical procedure and in the postoperative recovery room.

**Results:**

the number of patients analyzed was 78 with 39 patients in each group. There were no signification differences in demographic data and premedication acceptance between the two groups. Levels of anxiety before any premedication were similar in the two groups. However, the anxiety level 30 minutes after premedication and in the operating room was significantly lower in the clonidine group (p<0.001). Children who received clonidine showed better sedation on entering the operating room (p=0.002) as well as postoperatively on entering the post-anesthesia unit care (p=0.006). The hemodynamic and respiratory parameters recorded were statistically comparable.

**Conclusion:**

intranasal clonidine is an interesting premedication to prevent perioperative children´s anxiety with few side effects.

## Introduction

Up to 60% of children undergoing anesthesia and surgery can experience significant distress and anxiety during the perioperative period [1,2]. This clinical phenomenon is important since it may lead to psychological and physiological adverse outcomes [3]. It may prolong the induction of anesthesia and lead to maladaptive behavioral responses such as sleep and eating disturbances and enuresis [4,5]. Therefore, premedication before surgery is an important stage in anesthesia. The goals are to produce anxiolysis, sedation, amnesia, and analgesia. Benzodiazepines have been the pharmacological agent of choice for preoperative anxiety in daycare surgery because of their rapid onset and short half-life. However, it is far from an ideal premedication. Clonidine, an alpha2 adrenoceptor agonist, is gaining popularity among anesthesiologists. It lacks the majority of the negative effects associated with midazolam but its routine use is still limited because of its slow onset of action. This study aimed to evaluate intranasal clonidine as an anxiolytic premedication in pediatric anesthesia.

## Methods

We conducted a prospective controlled double-blinded randomized clinical trial in the anesthesia-intensive care unit involving children aged 2 to 15 years undergoing tonsillectomy surgery.

**Study population:** inclusion criteria were children aged between 2 and 15 years with an American Society of Anesthesiologists (ASA) score of I or II, who underwent elective tonsillectomy under general anesthesia. Non-inclusion criteria were any upper respiratory tract infection in the last 3 weeks, congenital heart disease, cardiac arrhythmias, intellectual disability, known allergy or hypersensitive reaction to clonidine or any excipient, organ dysfunction, long-term treatment with anxiolytic or anti-emetic. The exclusion criteria were non-respect of the study protocol.

**Sample size:** since there was no clinical trial investigating the effect of intranasal clonidine in the prevention of perioperative anxiety in children, the sample size was calculated from a previous pre-survey of 20 patients. A decrease of 15% in the m-YPAS scores 30 minutes after the administration of intranasal clonidine was considered significant. Calculation according to alpha at 5% and power of study at 90% produced a minimal sample size of 18 patients in each group. We then designated 40 patients per group to have sufficient numbers after possible exclusions.

**Randomization and allocation:** patients were randomized automatically according to a sequence generated by the website: sealedenvelope.com into two groups: P group (received 0.2 ml/kg of a 5 ml solution of physiological serum distributed over both nostrils) and C group (received 0.2 ml/kg of a 5 ml solution containing 20 μ g/ml of clonidine distributed over both nostrils).

**Intervention:** in the preoperative holding area, before any premedication, the anxiety level of the child was determined using an observational scale: m-YPAS (modified Yale perioperative anxiety scale). It is an observational measure of children’s preoperative anxiety consisting of 27 items divided into 5 categories: activity, vocalizations, emotional expressivity, state of arousal, and use of parent. Scores range from 22.5 to 100 with higher scores indicating greater anxiety.

Then according to the results of the randomization, premedication was given 30 minutes before anesthesia induction by an anesthesiologist who was blinded to the content of the solution. The drug acceptance by the children was recorded concerning the taste: good= if the patient was indifferent, average = if the patient makes a wince, and unpleasant= if the patient cries. At parents-separation, the anxiety level of the child was rated again using m-YPAS. In the operating room, the level of sedation was assessed by the World Health Organization´s (WHO) scale (S0: awake patient, S1: drowsy, verbally stimulable, S2: drowsy, tactile stimulable, S3: coma, not awake). Anxiety level was rated one last time using m-YPAS-without parent item. The heart rate, blood pressure, respiratory rate, and oxygen saturation were monitored continuously. Anesthesia was induced with inspired sevoflurane 8%. A 2-point scale was applied for the evaluation of mask acceptance: 1 = calm, cooperative, or easily calmed, 2 = not easily calmed, agitated, or angry. The emergence of sevoflurane-induced agitation was noted. After that, intravenous access was attained. Fentanyl (3 μg.kg^-1^), propofol (4 μg.kg^-1^) and atracurium (0.5 μg.kg^-1^) were administrated, and endotracheal intubation was performed.

Ringer´s lactate solution was infused according to the child´s weight. The maintenance of anesthesia was carried out with inspired sevoflurane 2.5% in a mixture of air 50% and oxygen 50%. Any hemodynamic change following intubation was recorded. Thirty minutes before the end of the intervention, a dose of 15 mg.kg^-1^ of paracetamol was infused. The child was extubated awake in the operating room after adequate neuromuscular recovery and when able to open his eyes. The time between stopping sedation and awakening was recorded as well as the occurrence of agitation. Then, the child was transferred to the recovery room. In the recovery room, standard monitoring was applied for 2 hours. The level of sedation was assessed by the WHO´s scale. The presence or not of agitation was noted. All the adverse effects including hypotension, bradycardia, respiratory depression, nausea/vomiting, and shivering were recorded.

**Data collection:** pre- and intraoperative data were collected including patients´ demographics, American Society of Anesthesiology (ASA) score, and duration of surgery. We collected in the pre-anesthetic phase: anxiolysis score before administration of the premedication: m-YPAS at time T1, acceptance of premedication: (good, average, or mediocre), and anxiolysis score at the time of parent-child separation: m-YPAS at time T2. We collected in the anesthetic phase: sedation score, anxiolysis score at installation in the operating room: m-YPAS truncated at time T3, the acceptance of the mask (good or bad), agitation on inhalation of sevoflurane (present or absent) or on extubation (present or absent), the wake-up time and the occurrence of other intraoperative incidents. We collected in the post-anesthetic phase: sedation score on arrival at the Post Interventional Monitoring Room (PIMR) and agitation (present or absent).

**Variables:** our primary outcome was to assess the efficacy of intranasal clonidine as premedication on the rate of anxiolysis at the time of parent-child separation and on admission to the operating room using the m-YPAS score. Our secondary outcomes were to assess the tolerance of intranasal administration of clonidine premedication, to evaluate the acceptability of the mask, the efficacy of intranasal clonidine on agitation on inhalation of anesthetic gas, on awakening and in Post Interventional Monitoring Room (PIMR), and the effect of clonidine on pre- and post-anesthetic sedation. We evaluate also the effect of clonidine on the prevention of postoperative nausea and vomiting (PONV) and postoperative chills.

**Statistical analysis:** SPSS version 25.0 software was used for data analysis. We checked the normality of the distribution by the Shapiro-Wilk test for the quantitative variables. Continuous variables and data with a normal distribution are expressed as means (SD) and as medians with the semi-interquartile ranges (SIR) otherwise. Qualitative variables were expressed as frequency distributions. Univariate comparisons between the two groups of patients were performed using t- student test, Mann-Whitney test, and Pearson´s Chi-square test. Statistical significance was defined as p<0.05.

**Ethical considerations:** this study was conducted after approval of the Southern Protection Committee of People (C.P.P.SUD) under the aegis of the Health Ministry of the Tunisian Republic reference CPP SUD N°16/2019 for the nature of the product and the conduct of the study. Written and informed parental consent was obtained.

## Results

**Figure 1:**
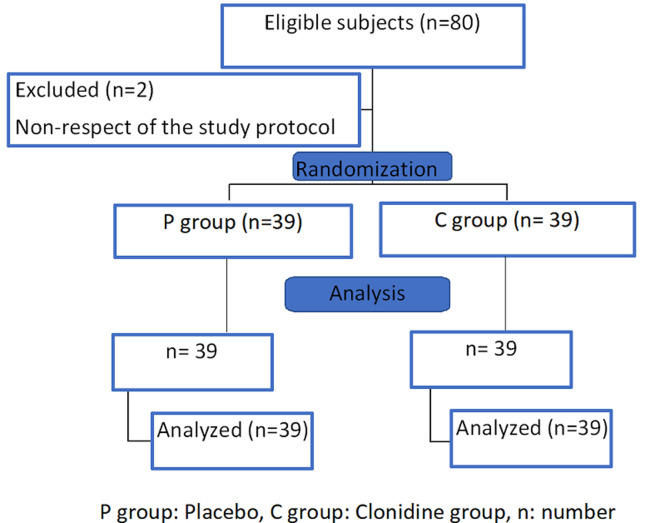
flow chart of study participants, recruited from the operating room of Habib Bourguiba Hospital Sfax Tunisia

Eighty children were included in our study and were divided into two equivalent groups. Two patients were excluded for non-respect of the study protocol. The number of patients analyzed was 78 with 39 patients in each group (Figure 1).

**General characteristics:** our sample was characterized by a mean age =5.91 ± 2.68 years, a sex ratio (male/female) =0.6 and a mean weight = 22.92 ± 10.38 kg. The two groups were comparable regarding age, gender, weight, and ASA (Table 1).

**Table 1:** comparison of demographic data between the two groups

Demographic data		P group (n=39)	C group (n=39)	p-value
Gender	Female (n)	11	18	0.080 ƚ
	Male (n)	28	21	
Age (years) ± SD		5.949 ± 2.901	5.872 ± 2.494	0.264 *
Weight (kg) ± SD		20.59 ±10.17	25.256 ± 10.176	0.993*
ASA (n)	ASA I	35	34	0.500 ƚ
	ASA II	4	5	

P group: placebo; C group: clonidine group; SD: standard deviation; n: effective; Kg: kilogram; *: t-student test, ƚ: Pearson´s Chi-square; ASA: American Society of Anesthesiologists

**Primary outcomes:** anxiolysis score m-YPAS before premedication (T1) was similar in the two groups (p=0.768). However, at the time of parent-child separation (T2) and in the operating room (T3), it was significantly decreased in the C group compared to the P group with p<0.001 (Table 2).

**Table 2:** comparison of anxiety score at different times between the two groups

Mean of m-YPAS	P group	C group	p-value
Mean of m-YPAS before premedication (T1) ± SD	51.09 ± 18.52	52.25 ± 15.94	0.768*
Mean of m-YPAS at parent-child separation (T2) ± SD	67.58 ± 26.1	36.04 ± 14.38	<0.001*
Mean of m-YPAS in the operating room (T3) ± SD	75.71 ± 28.7	33.86 ± 17.52	<0.001*

SD: standard-deviation; *: t- Student test; m-YPAS: modified Yale perioperative anxiety scale

**Secondary outcomes:** drug acceptance was good with placebo as well as with clonidine without no statistical difference between the two groups (p=0.807). In the operating room, all children of the P group were awake versus 19 children from the C group who were drowsy at different levels (S1 level: 18 children and S2 level: one child). This difference is statistically significant (p<0.001) (Table 3). Concerning mask acceptance, 9 patients from the P group accepted the mask versus 32 patients from the C group (p<0.001) (Table 3). The occurrence of agitation on inhalation of sevoflurane and extubation was significantly higher in the P group than in the C group with a p-value respectively <0.001 and 0.008 (Table 3). There were no statistical differences between the two groups concerning the wake-up time, hemodynamic modifications, and the occurrence of any other event (Table 3).

**Table 3:** comparison of per operative data between the two groups

Perioperative data		C group	P group	p-value
Sedation level	S0	20	39	< 0.001 ƚ
	S1	18	0	
	S2	1	0	
	S3	0	0	
Mask acceptance		32	9	< 0.001 ƚ
Sevoflurane induced agitation		6	32	< 0.001 ƚ
Agitation at extubation		7	18	0.008 ƚ
Tachycardia at intubation (n)		0	18	< 0.001 ƚ
Bradycardia (n)		0	1	0.128 ƚ
Hypotension (n)		2	2	0.695 ƚ
Desaturation (n)		0	1	0.128 ƚ
Bronchospasme (n)		1	0	0.128 ƚ
Wake-up time ± SD		13.10 ± 5.42	11.87 ± 4.23	0.386 *

P group: placebo; C group: clonidine group; n: effective; SD: standard-deviation; S0: awake patient; S1: drowsy, verbally stimulable; S2: drowsy, tactile stimulable; S3: coma, not awakening; *: t- Student test, ƚ: Pearson´s Chi-square

In the recovery room, there were no statistical differences between the two groups in terms of sedation score (Table 4). Patients from the C group presented less agitation (p=0.016), less nausea and vomiting (p=0.04), and less shivering (p<0.001) than the P group (Table 4).

**Table 4:** comparison of postoperative data between the two groups

Postoperative data		P group	C group	p-value
Sedation level	S0	22	18	0.298 ƚ
	S1	7	13	
	S2	10	8	
	S3	0	0	
Agitation in the recovery room		18	8	0.016 ƚ
Nausea and vomiting		4	0	0.04 ƚ
shivering		16	1	<0.001 ƚ

P group: placebo; C group: Clonidine group; S0: awake patient; S1: drowsy, verbally stimulable; S2: drowsy, tactile stimulable; S3: coma, not awakening; ƚ: Pearson´s Chi-square

## Discussion

We conducted a prospective randomized clinical trial to evaluate the efficacity of clonidine in reducing perioperative children´s anxiety. Our study demonstrates that intranasal clonidine is effective in preoperative anxiety, preoperative and postoperative sedation, quality of induction and intubation, quality of awakening after anesthesia, and quality of immediate postoperative period compared to intranasal placebo when it is used as a premedication in children proposed for tonsillectomy.

Clonidine is in increasing use in the pediatric population as an anxiolytic, sedative, and analgesic because of its central alpha2-adrenergic agonist effect [6-12]. However, one major drawback of clonidine used as an anxiolytic premedicant is its slow onset of action when used orally [13]. So, intranasal clonidine was first tested in rodents by Babhair *et al*. [14] who showed a rapid onset time of action within 10 minutes without increased side effects. Studies have shown similar bioavailability close to 100% with rapid and non-invasive administration of the intranasal route in children [15-18]. The use of the modified Yale Preoperative Anxiety Scale (mYPAS) in this study allowed us to evaluate the changes in anxiety levels and differentiate the sedative effect from the anxiolytic effect. The first investigations carried out on clonidine as premedication in children were carried out by Mikawa *et al*. [8]. Clonidine was administered orally at two different doses 4 μg/kg and 2 μg/kg. The results showed a dose-dependent preoperative anxiolytic effect without an increase in side effects. In our study, the number of children who achieved satisfying anxiolysis was larger with intranasal clonidine compared to placebo with a statistically significant difference. Several studies [17-19] have compared clonidine to midazolam, which was the gold standard for premedication in pediatric surgery. The results prove a higher tolerance for clonidine because of the absence of nasal mucosa irritation and its neutral taste. In our study, we compared clonidine with placebo in terms of acceptance, we did not find a statistically significant difference between the two solutions administered intranasally with a satisfactory acceptance rate exceeding 50%.

Clonidine is responsible for sedation close to physiological sleep from which the patient can be easily awakened and in possession of his cognitive faculties. It has even been shown that once awake, patients are quite capable of carrying out psychomotor tests [20,21]. In our study, 61.29% of the children in the clonidine group were drowsy on entering the operating room without any significant difference between the two groups in terms of delay in awakening and sedation on arrival in the recovery room. Clonidine improves the quality of an inhalator induction of general anesthesia by making it easier and calmer [9,15,18].

All these studies have shown that patients who received clonidine premedication accepted the face mask better than those who received another molecule, thanks to the anxiolytic effect provided by clonidine. Post-anesthetic agitation is a common problem in children who have undergone general anesthesia with sevoflurane. This agitation is characterized by a change in the child's perception of his environment with signs of disorientation, hypersensitivity to stimuli, and hyperactivity. Our study showed that intranasal clonidine reduced significantly postoperative agitation. Zhang *et al*. [22] found that both the sedative and analgesic effects of clonidine reduce post-anesthetic agitation in the pediatric population. Postoperative nausea and vomiting (PONV) are one of the complications associated with surgery. Our trial demonstrated that children with intranasal clonidine presented less PONV than placebo. A meta-analysis, published by Dahmani *et al*. [23], studied three clinical trials [24-26] which all compared the effect of oral clonidine versus diazepam for the prevention of PONV. This meta-analysis concluded that the effect of clonidine was superior to diazepam. The occurrence of shivering is observed in 5 to 65% of patients in the postoperative period [11].

In our study, we noted a single case of postoperative shivering in the clonidine group versus 16 children in the placebo group with a significant difference (p<0.001). A Cochrane meta-analysis [27] demonstrated a reduction in the incidence of postoperative shivering by alpha2-agonists, whether clonidine or dexmedetomidine. Dosages and routes of administration used were varied but the difference was always significant, with an intermediate level of evidence [27]. Initially known for its antihypertensive effects, clonidine, an alpha2 agonist sees its place still subject to debate mainly in the pediatric population for fear of hemodynamic side effects. However, the incidents reported in the literature during premedication with clonidine were very rare at the recommended doses [19,28].

Our study has several limitations such as the choice of the sedation evaluation scale. We used it for the evaluation of the sedation on the WHO sedation scale. However, it is a scale applicable after the use of opioid products and whose validity in young patients is still limited.

## Conclusion

Intranasal clonidine administration is an interesting alternative as an anxiolytic premedication before pediatric surgery. It produces effective sedation with few side effects.

### What is known about this topic

- *Anxiety in children during the perioperative period may lead to psychological and physiological adverse outcomes;*
- *Benzodiazepines such as midazolam have been the pharmacological agent of choice for preoperative anxiety in daycare surgery;*
- *Clonidine is an alpha2 adrenoceptor agonist and has an anxiolytic, sedative, and analgesic effect*.

### What this study adds

- *Intranasal clonidine prevents perioperative children´s anxiety;*
- *Intranasal clonidine provides less agitation and shivering in children;*
- *Intranasal clonidine provides less nausea and vomiting in children*.

## References

[1] Sola C, Lefauconnier A, Bringuier S, Raux O, Capdevila X, Dadure C (2017). Childhood preoperative anxiolysis: Is sedation and distraction better than either alone? A prospective randomized study. Paediatr Anaesth.

[2] Kain ZN, Mayes LC, O´Connor TZ, Cicchetti DV (1996). Preoperative anxiety in children. Predictors and outcomes. Arch Pediatr Adolesc Med.

[3] West N, Christopher N, Stratton K, Görges M, Brown Z (2020). Reducing preoperative anxiety with Child Life preparation prior to intravenous induction of anesthesia: A randomized controlled trial. Paediatr Anaesth.

[4] Amouroux R, Rousseau-Salvador C, Annequin D (2010). L´anxiété préopératoire: manifestations cliniques, évaluation et prévention. Ann Méd-Psychol Rev Psychiatr.

[5] Kain ZN, Mayes LC, Caldwell-Andrews AA, Karas DE, McClain BC (2006). Preoperative anxiety, postoperative pain, and behavioral recovery in young children undergoing surgery. Pediatrics.

[6] Carabine UA, Wright PMC, Moore J (1991). Preanaesthetic medication with clonidine: a dose-response study. Br J Anaesth.

[7] Wright PM, Carabine UA, McClune S, Orr DA, Moore J (1990). Preanaesthetic medication with clonidine. Br J Anaesth.

[8] Mikawa K, Maekawa N, Nishina K, Takao Y, Yaku H, Obara H (1993). Efficacy of oral clonidine premedication in children. Anesthesiology.

[9] Mikawa K, Nishina K, Shiga M (2002). Prevention of sevoflurane-induced agitation with oral clonidine premedication. Anesth Analg.

[10] Buggy D, Higgins P, Moran C, O'Donovan F, McCarroll M (1997). Clonidine at induction reduces shivering after general anaesthesia. Can J Anaesth.

[11] Kranke P, Eberhart LHJ, Roewer N, Tramer MR (2003). Postoperative shivering in children: a review on pharmacologic prevention and treatment. Pediatr Drugs.

[12] Alizadeh R, Mireskandari SM, Azarshahin M, Darabi ME, Padmehr R, Jafarzadeh A (2012). Oral clonidine premedication reduces nausea and vomiting in children after appendectomy. Iran J Pediatr.

[13] Nishina K, Mikawa K (2002). Clonidine in paediatric anaesthesia. Curr Opin Anaesthesiol.

[14] Babhair SA, Tariq M, Abdullah ME (1990). Comparison of intravenous and nasal bioavailability of clonidine in rodents. Res Commun Chem Pathol Pharmacol.

[15] Almenrader N, Passariello M, Coccetti B, Haiberger R, Pietropaoli P (2007). Steal-induction after clonidine premedication: a comparison of the oral and nasal route. Paediatr Anaesth.

[16] Larsson P, Eksborg S, Lönnqvist PA (2012). Onset time for pharmacologic premedication with clonidine as a nasal aerosol: a double-blind, placebo-controlled, randomized trial. Paediatr Anaesth.

[17] Stella MJ, Bailey AG (2008). Intranasal clonidine as a premedicant: three cases with unique indications. Paediatr Anaesth.

[18] Mitra S, Kazal S, Anand LK (2014). Intranasal clonidine vs. midazolam as premedication in children: a randomized controlled trial. Indian Pediatr.

[19] Kogan A, Katz J, Efrat R, Eidelman LA (2002). Premedication with midazolam in young children: a comparison of four routes of administration. Pediatr Anesth.

[20] Jatti K, Batra YK, Bhardwaj N, Malhotra S (1998). Comparison of psychomotor functions and sedation following premedication with oral diazepam and clonidine in children. Int J Clin Pharmacol Ther.

[21] Hall JE, Uhrich TD, Ebert TJ (2001). Sedative, analgesic and cognitive effects of clonidine infusions in humans. Br J Anaesth.

[22] Zhang C, Li J, Zhao D, Wang Y (2013). Prophylactic midazolam and clonidine for emergence from agitation in children after emergence from sevoflurane anesthesia: a meta-analysis. Clin Ther.

[23] Dahmani S, Brasher C, Stany I, Golmard J, Skhiri A, Bruneau B (2010). Premedication with clonidine is superior to benzodiazepines. A meta analysis of published studies. Acta Anaesthesiol Scand.

[24] Fazi L, Jantzen EC, Rose JB, Kurth CD, Watcha MF (2001). A comparison of oral clonidine and oral midazolam as preanesthetic medications in the pediatric tonsillectomy patient. Anesth Analg.

[25] Mikawa K, Nishina K, Maekawa N, Asano M, Obara H (1995). Oral clonidine premedication reduces vomiting in children after strabismus surgery. Can J Anaesth.

[26] Handa F, Fujii Y (2001). The efficacy of oral clonidine premedication in the prevention of postoperative vomiting in children following strabismus surgery. Paediatr Anaesth.

[27] Lewis SR, Nicholson A, Smith AF, Alderson P (2015). Alpha-2 adrenergic agonists for the prevention of shivering following general anaesthesia. Cochrane Database Syst Rev.

[28] Sabourdin N, Constant I (2009). Prémédication à la clonidine chez l´enfant. Prat En Anesth Réanimation.

